# 1,5-Anhydro-D-fructose Protects against Rotenone-Induced Neuronal Damage In Vitro through Mitochondrial Biogenesis

**DOI:** 10.3390/ijms22189941

**Published:** 2021-09-14

**Authors:** Yuki Kasamo, Kiyoshi Kikuchi, Munekazu Yamakuchi, Shotaro Otsuka, Seiya Takada, Yuki Kambe, Takashi Ito, Ko-ichi Kawahara, Kazunori Arita, Koji Yoshimoto, Ikuro Maruyama

**Affiliations:** 1 Department of Neurosurgery, Kagoshima University Graduate School of Medical and Dental Science, Kagoshima 890-8520, Japan; k1046118@kadai.jp (Y.K.); karita@m2.kufm.kagoshima-u.ac.jp (K.A.); kyoshimo@m.kufm.kagoshima-u.ac.jp (K.Y.); 2 Department of Systems Biology in Thromboregulation, Kagoshima University Graduate School of Medical and Dental Science, Kagoshima 890-8520, Japan; k3360022@kadai.jp (S.O.); karaagetantou0110@gmail.com (S.T.); takashi@m3.kufm.kagoshima-u.ac.jp (T.I.); koichi.kawahara@oit.ac.jp (K.-i.K.); 3 Department of Physiology, Division of Brain Science, Kurume University School of Medicine, Kurume 830-0011, Japan; 4 Department of Laboratory and Vascular Medicine, Kagoshima University Graduate School of Medical and Dental Science, Kagoshima 890-8520, Japan; munekazu@m.kufm.kagoshima-u.ac.jp; 5 Department of Pharmacology, Kagoshima University Graduate School of Medical and Dental Science, Kagoshima 890-8544, Japan; ukikambe@m3.kufm.kagoshima-u.ac.jp; 6 Laboratory of Functional Foods, Department of Biomedical Engineering, Osaka Institute of Technology, Osaka 535-8585, Japan

**Keywords:** 1,5-AF, 1,5-AG, metformin, Parkinson’s disease, parkinsonism, AMPK, PGC-1α, mitochondria, mitochondrial biogenesis, rotenone

## Abstract

Mitochondrial functional abnormalities or quantitative decreases are considered to be one of the most plausible pathogenic mechanisms of Parkinson’s disease (PD). Thus, mitochondrial complex inhibitors are often used for the development of experimental PD. In this study, we used rotenone to create in vitro cell models of PD, then used these models to investigate the effects of 1,5-anhydro-D-fructose (1,5-AF), a monosaccharide with protective effects against a range of cytotoxic substances. Subsequently, we investigated the possible mechanisms of these protective effects in PC12 cells. The protection of 1,5-AF against rotenone-induced cytotoxicity was confirmed by increased cell viability and longer dendritic lengths in PC12 and primary neuronal cells. Furthermore, in rotenone-treated PC12 cells, 1,5-AF upregulated peroxisome proliferator-activated receptor-γ coactivator 1α (PGC-1α) expression and enhanced its deacetylation, while increasing AMP-activated protein kinase (AMPK) phosphorylation. 1,5-AF treatment also increased mitochondrial activity in these cells. Moreover, PGC-1α silencing inhibited the cytoprotective and mitochondrial biogenic effects of 1,5-AF in PC12 cells. Therefore, 1,5-AF may activate PGC-1α through AMPK activation, thus leading to mitochondrial biogenic and cytoprotective effects. Together, our results suggest that 1,5-AF has therapeutic potential for development as a treatment for PD.

## 1. Introduction

Parkinson’s disease (PD) is one of the most common neurodegenerative diseases, especially among older individuals. Because the aged population is gradually increasing worldwide, treatments that inhibit the progression of this disease are increasingly important [1,2]. The causes and pathomechanisms of PD remain largely unknown; however, it has been postulated that intrinsic or extrinsic neurotoxic metabolites or substances participate in its pathogenesis [3]. PD is strongly linked to mitochondrial dysfunction in terms of both environmental risk factors (e.g., exposure to many pesticides [4] and some metals [5]) and genetic risk factors (e.g., *PARK2* and *PARK6* [6]). Additionally, rotenone and other substances that inhibit the mitochondrial electron transport chain may have roles in developing PD [7,8,9]. Rotenone is a mitochondrial complex I inhibitor that induces apoptosis by increasing mitochondrial reactive oxygen species [10]. Rotenone is often used to create animal and cell models to study the pathomechanisms of PD; it is also used to aid in developing treatments and interventions for PD [11]. In addition, the relationship between PD and peroxisome proliferator-activated receptor-γ coactivator 1α (PGC-1α), a protein involved in mitochondrial biogenesis, has been demonstrated in recent years. Zheng et al. reported that the expression of PGC-1α-regulated genes is decreased in many PD patients [12], while Siddiqui et al. demonstrated that increased PGC-1α expression protects against α-synuclein toxicity [13]. Furthermore, O’Donnell et al. reported that PGC-1α protects axons from α-synuclein toxicity in a zebrafish model [14].

PGC-1α is a transcriptional coactivator that binds to and co-activates transcription factors, leading to mitochondrial biogenesis. The process of mitochondrial biogenesis is complex, from the transcription of genes related to mitochondria to increased intracellular mitochondrial components and energy capacity. Little is known about how mitochondrial biogenesis might play a role in the pathology of PD. However, it is expected that models induced by neurotoxins, such as rotenone, will provide clues to the relationship between PD and mitochondrial biogenesis.

**Figure 1:**
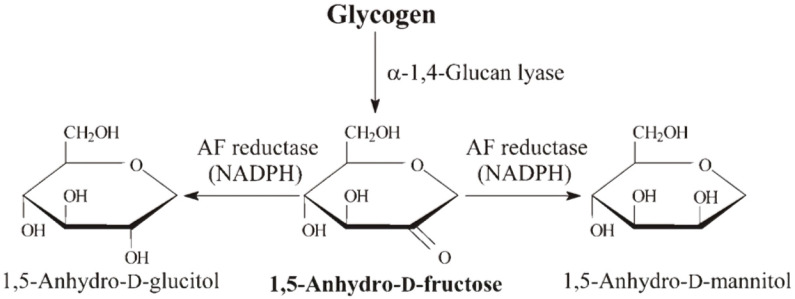
Chemical structure of 1,5-AF and related enzymatic reactions. 1,5-AF, 1,5-anhydro-D-fructose.

1,5-anhydro-D-fructose (1,5-AF) is a monosaccharide that is directly formed from starch and glycogen in food by the action of α-1,4-glucan lyase (EC 4.2.2.13) in the liver [15,16,17]. It is then reduced to 1,5-anhydro-D-glucitol (1,5-AG) and reabsorbed in the kidney. 1,5-AG is usually maintained at a constant level in the blood. It is used clinically as a marker of glucose metabolism, and its blood concentration is decreased in poorly controlled diabetic patients [18]. Although 1,5-AG is not thought to be used as an energy source, blood levels of 1,5-AG are maintained in a constant range; however, the reason for this remains unknown. The chemical structure of 1,5-AF and brief summaries of related enzymatic reactions are shown in Figure 1.

We have previously reported the cytoprotective effects of 1,5-AF against a range of proinflammatory and cytotoxic substances, including lipopolysaccharides [17,19]. We also have data suggesting that 1,5-AF activates AMP-activated protein kinase (AMPK) (unpublished data). Furthermore, it has been reported that metformin, which is currently the most popular antidiabetic medicine, also activates AMPK, leading to mitochondrial biogenic activity via PGC-1α [20].

We, therefore, used rotenone to create cell models of PD and investigated the effects of 1,5-AF in these models. Based on these observations, we then examined the cytoprotective effects of 1,5-AF on rotenone-induced cytotoxicity in vitro using cultured neuronal cells. We demonstrated that 1,5-AF had cytoprotective and mitochondrial biogenic effects. Together, our results suggest that 1,5-AF may have therapeutic potential for development as a treatment for PD.

## 2. Results

### 2.1. 1,5-AF Ameliorates Rotenone-Induced Cytotoxic Effects in a Dose-Dependent Manner

**Figure 2:**
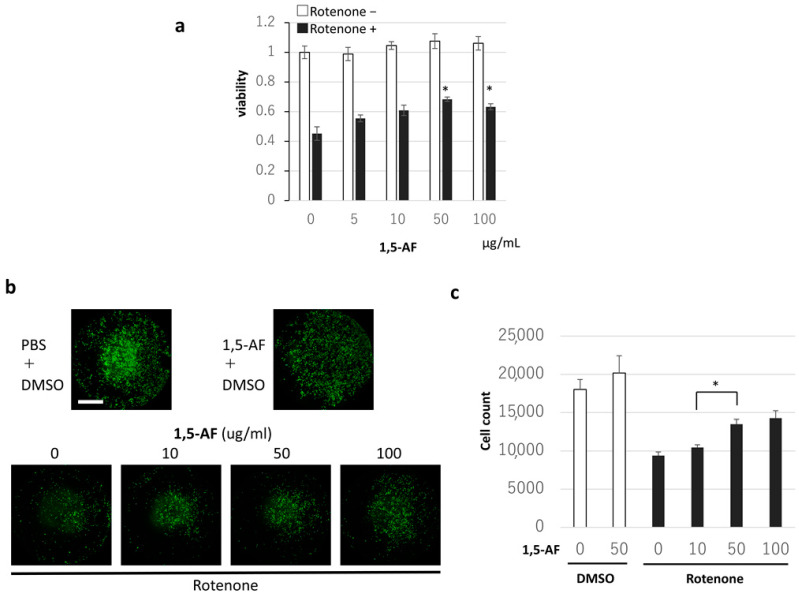
Effects of 1,5-AF against rotenone-induced cytotoxicity in cultured PC12 cells. (**a**–**c**) Cells were incubated with control solvent (DMSO) or rotenone for 24 h; viable cells were then counted to evaluate the cytotoxic effects of rotenone in this cell line. (**a**) The addition of 1,5-AF had cytoprotective activity against rotenone-induced cytotoxic effects when evaluated by MTT assay (two-way ANOVA). (**b**) Representative confocal images of calcein AM-stained cells showing the dose-dependent cytoprotective effects of 1,5-AF against rotenone (magnification: 4×; scale bar: 2000 µm). (**c**) Quantification of the dose-dependent cytoprotective effects of 1,5-AF from the confocal images of calcein AM-stained cells (one-way ANOVA). Data represent mean cell counts using BZ-X Analyzer software. All data are expressed as the mean ± standard error of the mean. * *p* < 0.05. 1,5-AF, 1,5-anhydro-D-fructose; DMSO, dimethyl sulfoxide; PBS, phosphate-buffered saline.

**Figure 3:**
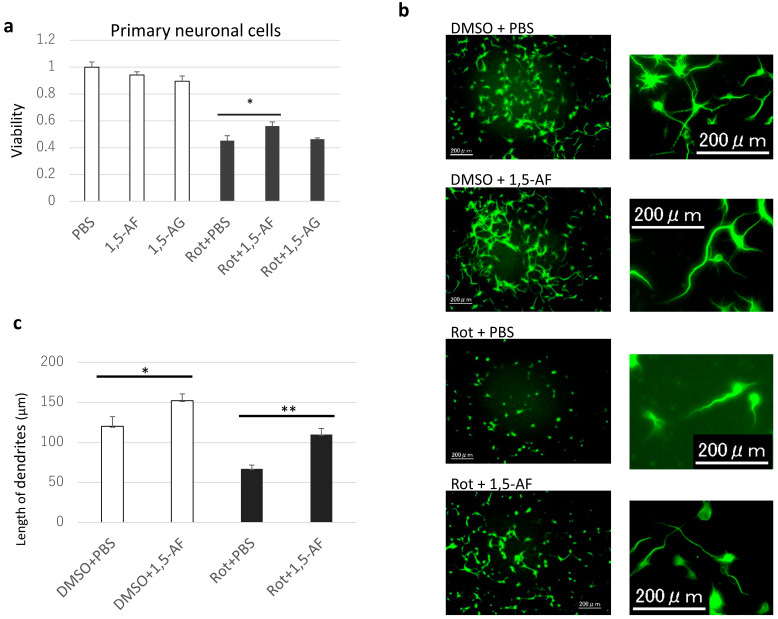
Protective effects of 1,5-AF against rotenone in primary neuronal cells. (**a**) Protective effects of 1,5-AF against rotenone-induced cytotoxicity in primary neuronal cells, evaluated using the MTT assay (one-way analysis of variance). (**b**) Representative confocal images of microtubule-associated protein 2 (MAP2) staining in primary neuronal cells (magnification: 20×; scale bars: 200 µm). Panels in the right column are magnified versions of images in the left column. Rotenone affected cell shape, dendrite length, and cell number. (**c**) Quantification of the effects of 1,5-AF on dendritic length from confocal images of MAP2-stained primary neuronal cells. Rotenone affected dendritic length. All data are expressed as the mean ± standard error of the mean. * *p* < 0.05, ** *p* < 0.01. 1,5-AF, 1,5-anhydro-D-fructose; 1,5-AG, 1,5-anhydroglucitol; DMSO, dimethyl sulfoxide; PBS, phosphate-buffered saline; Rot, rotenone.

Increasing concentrations of rotenone caused dose-dependent decreases in cell viability (Supplementary Figure S1). Because 50% of PC12 cells survived at a rotenone concentration of 1 µM, PC12 cells were stimulated with rotenone at 1 µM for 24 h. The MTT assay results demonstrated that when 1,5-AF was added in the range of 0–100 μg/mL, cell viability (average ± SD) was 68 ± 1% and 63 ± 2% with 1,5-AF concentrations of 50 μg/mL and 100 μg/mL, respectively, compared with 45 ± 5% in the control group, indicating that rotenone significantly prevented cell death (*p* = 0.001 and *p* = 0.015, respectively; two-way ANOVA; Figure 2a). In addition, cell imaging using calcein AM staining (Figure 2b,c) revealed that 50 µg/mL 1,5-AF significantly inhibited rotenone-induced cell death compared with 10 µg/mL 1,5-AF in terms of cell counts (average ± SD; 13,508 ± 619 cells and 10,466 ± 334 cells, respectively; *p* = 0.004). These findings indicate that 1,5-AF tends to inhibit rotenone-induced cell death in a dose-dependent manner. The protective effects of 1,5-AF against 50 nM rotenone-induced cell death over 24 h (*p* < 0.05, compared with rotenone + phosphate-buffered saline (PBS) treatment) were also confirmed in mouse primary neuronal cells (Figure 3a). These results suggest that 1,5-AF has an inhibitory effect on rotenone-induced cytotoxicity.

Additionally, dendritic elongation (*p* < 0.05, compared with dimethyl sulfoxide (DMSO) + PBS treatment) and a neuroprotective effect of 1,5-AF against rotenone-induced dendrite shortening (*p* < 0.01, compared with rotenone + PBS treatment) were confirmed in primary neuronal cells (Figure 3b,c).

**Figure 4:**
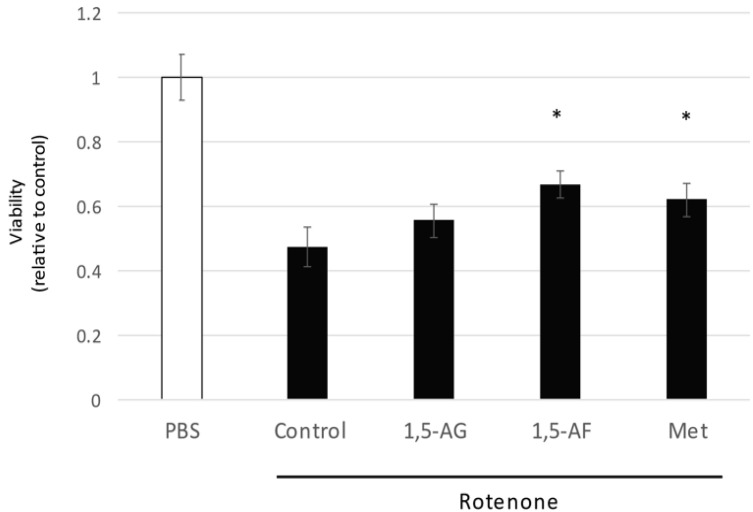
Effects of 1,5-AF, 1,5-AG, and metformin on rotenone-induced cytotoxicity in PC12 cells. Both 1,5-AF and metformin, but not 1,5-AG, had protective effects against rotenone-induced cytotoxicity, evaluated using the MTT assay (one-way analysis of variance followed by Dunnett’s test). Data are expressed as the mean ± standard error of the mean of quadruplicate pooled experiments (*n* = 8 for the Control, PBS, and 1,5-AF conditions; *n* = 4 for the Met and 1,5-AG conditions). * *p* < 0.05. 1,5-AF, 1,5-anhydro-D-fructose; 1,5-AG, 1,5-anhydroglucitol; Met, metformin; PBS, phosphate-buffered saline; Rot, rotenone.

The 1,5-AF metabolite 1,5-AG did not show cytoprotective effects against rotenone treatment (Figure 3a and Figure 4). However, another compound known to exhibit mitochondrial protective activity, metformin [20], showed cytoprotective effects similar to the effects of 1,5-AF (*p* < 0.05 for both 1,5-AF and metformin, compared with control; *p* = 0.29 for comparison between 1,5-AF and metformin; Figure 4). These findings indicated that the effects of 1,5-AF were not elicited by its metabolite (1,5-AG) despite the structural similarities; moreover, the effects of 1,5-AF were similar to the mitochondria-specific effects of a compound known to protect mitochondria (metformin).

### 2.2. 1,5-AF Upregulates PGC-1α Expression and Deacetylation While Increasing Phosphorylated AMPK

**Figure 5:**
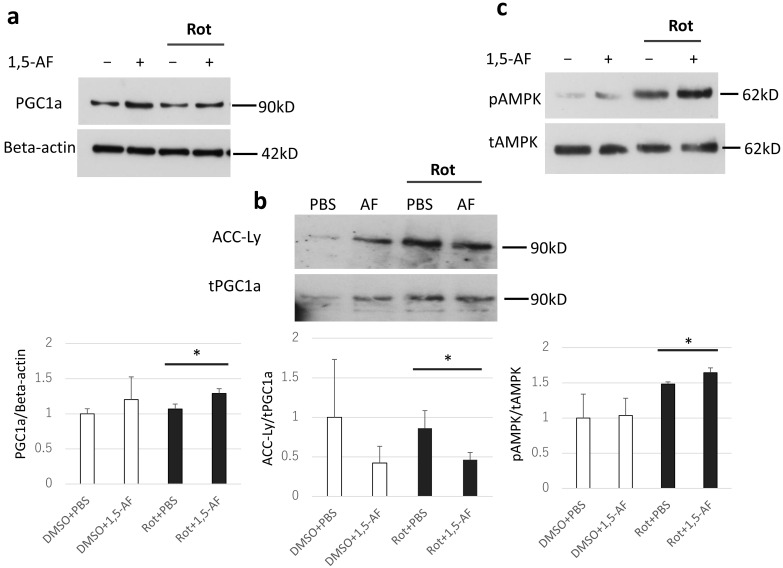
Immunoblotting and quantitative immunoprecipitation evaluated the effects of 1,5-AF treatment on PGC-1α and AMPK proteins and activities in rotenone-treated PC12 cells. (**a**) Evaluation of PGC-1α protein level by immunoblotting. In rotenone-treated cells, PGC-1α protein expression was increased by 1,5-AF treatment. (**b**) Evaluation of PGC-1α acetylation assays. In rotenone-treated cells, deacetylated PGC-1α was increased by 1,5-AF treatment. (**c**) Total AMPK and phosphorylated AMPK were evaluated by immunoblotting. In rotenone-treated cells, the ratio of phosphorylated AMPK to total AMPK was increased by 1,5-AF treatment. All data are expressed as the mean ± standard error of the mean of quadruplicate experiments. * *p* < 0.05. 1,5-AF, 1,5-anhydro-D-fructose; ACC-Ly, acetylated lysine; DMSO, dimethyl sulfoxide; Met, metformin; pAMPK, phosphorylated AMP-activated protein kinase; PBS, phosphate-buffered saline; PGC-1α, peroxisome proliferator-activated receptor-γ coactivator 1α; Rot, rotenone; tAMPK, total AMP-activated protein kinase; tPGC1a, total PGC-1α. Note: tPGC1a in panel (**b**) represents PGC-1α in samples immunoprecipitated with anti-PGC-1α antibody, whereas PGC1a in panel (**a**) represents PGC-1α in cell lysates that were not subjected to immunoprecipitation.

We investigated the mechanism by which 1,5-AF protects against rotenone. As shown in Figure 5a, 1,5-AF treatment increased PGC-1α expression in rotenone-stimulated PC12 cells (*p* < 0.05, compared with rotenone + PBS treatment). Additionally, in the DMSO cell group (no rotenone treatment), 1,5-AF treatment appeared to increase the expression of PGC-1α protein, although this trend was not significant. Figure 5b shows an analysis of immunoprecipitation with anti-PGC-1α antibody followed by immunoblotting of the sample with acetylated lysine (ACC-Ly) and total PGC-1α, respectively. Based on previous reports that PGC-1α is activated by deacetylation [21], an increase in ACC-Ly was taken to mean a decrease in activated PGC-1α. 1,5-AF treatment resulted in a reduced ratio of ACC-Ly to total PGC-1α (*p* < 0.05, compared with rotenone + PBS treatment), which implied increased deacetylation of PGC-1α (Figure 5b). Our findings suggest that the ratio of activated PGC-1α was increased by 1,5-AF treatment in rotenone-stimulated PC12 cells. Notably, 1,5-AF alone also tended to increase the expression and activation of PGC-1α. Finally, 1,5-AF treatment increased the ratio of phosphorylated AMPK (i.e., activated AMPK) to total AMPK in rotenone-stimulated PC12 cells (*p* < 0.05, compared with rotenone + PBS treatment; Figure 5c). Overall, our findings suggest that 1,5-AF may activate PGC-1α in an AMPK-dependent manner in rotenone-stimulated PC12 cells.

### 2.3. PGC-1α Silencing Inhibits 1,5-AF-Mediated Protection against Rotenone-Induced Cytotoxicity

**Figure 6:**
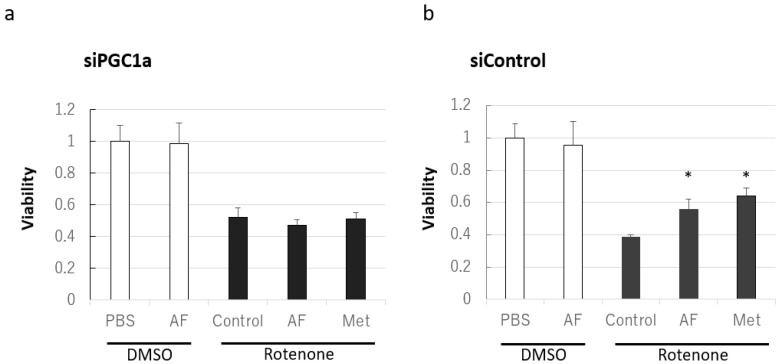
Effects of PGC-1α silencing on rotenone-induced cytotoxicity in cultured PC12 cells treated with 1,5-AF or metformin. (**a**) Silencing of PGC-1α by *PPARGC1A* small interfering RNA (siRNA) attenuated the protective effects of 1,5-AF and metformin on rotenone-induced cytotoxicity, evaluated using the MTT assay (one-way ANOVA followed by Dunnett’s test). (**b**) 1,5-AF and metformin protect against rotenone-induced cytotoxicity in negative control siRNA-transfected cells, evaluated using the MTT assay (one-way ANOVA). All data are expressed as the mean ± standard error of the mean. * *p* < 0.05. AF, 1,5-anhydro-D-fructose; DMSO, dimethyl sulfoxide; Met, metformin; PBS, phosphate-buffered saline; PGC-1α, peroxisome proliferator-activated receptor-γ coactivator 1α.

Figure 6 shows that in the PC12 cells transfected with *PPARGC1A* small interfering RNA (siRNA), which silences PGC-1α expression (Supplementary Figure S2), 1,5-AF did not have a cytoprotective effect (Figure 6a). In contrast, in negative control siRNA-transfected cells, 1,5-AF had a significant cytoprotective effect against rotenone (*p* < 0.05, compared with control; Figure 6b). These results indicate that PGC-1α silencing inhibits 1,5-AF-mediated protection against rotenone-induced cytotoxicity. In addition, the cytoprotective effects of metformin against rotenone were inhibited by the transfection of *PPARGC1A* siRNA (Figure 6a), while metformin showed a significant cytoprotective effect against rotenone in negative control siRNA-transfected cells (*p* < 0.05, compared with control; Figure 6b); these findings were consistent with previous reports that metformin-mediated protection involves PGC-1α activity [22,23].

### 2.4. 1,5-AF Increases MitoTracker Intensity and Activity of Mitochondria

**Figure 7:**
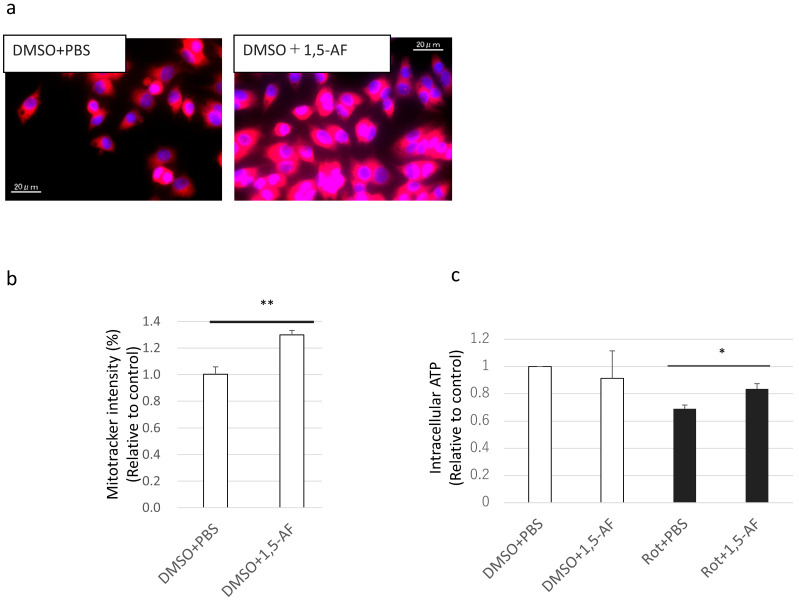
Effects of 1,5-AF treatment on mitochondrial quantity and quality in cultured PC12 cells. (**a**) Representative confocal images of MitoTracker staining (magnification: 100×; scale bar: 20 µm). (**b**) Quantification of the effects of 1,5-AF treatment on MitoTracker intensity from confocal images of MitoTracker-stained cells. (**c**) Effects of 1,5-AF treatment on intracellular ATP levels. All data are expressed as the mean ± standard error of the mean. * *p* < 0.05, ** *p* < 0.01. 1,5-AF, 1,5-anhydro-D-fructose; DMSO, dimethyl sulfoxide; PBS, phosphate-buffered saline; Rot, rotenone.

To assess the effects of 1,5-AF treatment on mitochondria, we performed mitochondrial staining and intracellular ATP measurements in PC12 cells. MitoTracker was used for mitochondrial staining. The import of MitoTracker into mitochondria depends on many factors, e.g., mitochondrial mass, number of mitochondria per cell, and mitochondrial membrane potential. 1,5-AF significantly increased intracellular MitoTracker intensity (*p* < 0.01; Figure 7a,b). In addition, under rotenone stimulation, MitoTracker intensity demonstrated a relative increase after 1,5-AF administration (Supplementary Figure S3); however, this change was unable to be evaluated absolutely because rotenone may decrease MitoTracker intensity by decreasing the membrane potential. Furthermore, 1,5-AF significantly increased intracellular ATP levels under conditions of rotenone exposure (*p* < 0.05; Figure 7c). These results indicate that 1,5-AF treatment increased MitoTracker intensity per cell with or without rotenone administration and suppressed the decrease in mitochondrial activity caused by rotenone exposure.

### 2.5. PGC-1α Silencing Inhibits the Effects of 1,5-AF on Mitochondria

**Figure 8:**
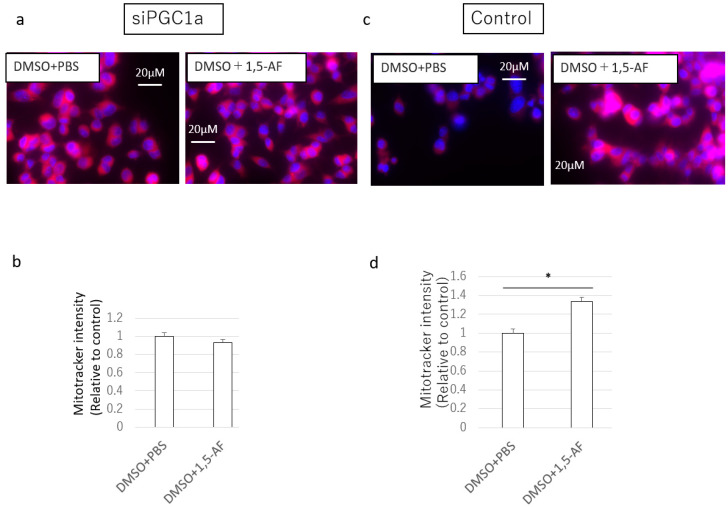
Effects of PGC-1α silencing on the mitochondrial protective activity of 1,5-AF in cultured PC12 cells. (**a**) Representative confocal images of MitoTracker staining in cells transfected with *PPARGC1A* small interfering RNA (siRNA; magnification: 100×, scale bar: 20 µm). (**b**) Transfection with *PPARGC1A* siRNA inhibited the increase in MitoTracker intensity of 1,5-AF-treated cells. (**c**) Representative confocal images of MitoTracker staining in cells transfected with control siRNA (magnification: 100×; scale bar: 20 µm). (**d**) In cells transfected with control siRNA, treatment with 1,5-AF increased the MitoTracker intensity in DMSO-treated cells. All data are expressed as the mean ± standard error of the mean. * *p* < 0.05. 1,5-AF, 1,5-anhydro-D-fructose; DMSO, dimethyl sulfoxide; PBS, phosphate-buffered saline; siPGC1a, cells transfected with *PPARGC1A* siRNA.

To assess whether PGC-1α is a crucial molecule for the effects of 1,5-AF treatment in increasing MitoTracker intensity, we silenced PGC-1α in PC12 cells. As shown in Figure 8, in PC12 cells transfected with *PPARGC1A* siRNA, 1,5-AF did not increase MitoTracker intensity per cell (Figure 8a,b). In contrast, in the control group, 1,5-AF significantly increased MitoTracker intensity per cell (*p* < 0.05; Figure 8c,d). Even with rotenone treatment, the 1,5-AF-induced increase in MitoTracker intensity was canceled in PC12 cells transfected with *PPARGC1A* siRNA (Supplementary Figure S4). Together, these results suggest that transfection with *PPARGC1A* siRNA inhibited the effects of 1,5-AF on MitoTracker intensity per cell.

## 3. Discussion

The monosaccharide 1,5-AF that was used in the present study is constantly produced from starch in very tiny amounts in the liver through the action of α-1,4-glucan lyase [15,16,17,19]. This 1,5-AF is rapidly reduced to 1,5-AG, which can be used as a urinary marker of renal function because 1,5-AG is reabsorbed from glucose transporter type 4 (GLUT-4) [24]. However, the physiological functions of 1,5-AF and 1,5-AG remain unknown. We therefore investigated the physiological functions of 1-5-AF. We have previously succeeded in producing 1,5-AF in vitro from sweet potatoes [24]. In the present study, we demonstrated that this 1,5-AF had cytoprotective effects that were exerted by protecting mitochondria from the mitochondrial toxin rotenone, which causes parkinsonism. Based on these data, we propose that 1,5-AF may have therapeutic potential for the treatment of PD.

In the current study, the addition of increasing amounts of 1,5-AF prevented rotenone-induced cell death in a dose-dependent manner in the PC12 cell line and primary neuronal cells. The cytoprotective activity of 1,5-AF against rotenone was also confirmed by cellular imaging using calcein AM staining and through assessments of dendritic length in cultured primary neuronal cells. There are two possible interpretations regarding these dendritic changes. The first possibility is that the promotion of mitochondrial function enhances that dendrite formation. This possibility is supported by our finding that 1,5-AF increased the length of microtubule-associated protein 2 (MAP2)-positive spines in neurons without rotenone treatment. Similarly, Li et al. reported [25] that mitochondrial activation increases dendritic spine length. The second possibility is that 1,5-AF inhibits rotenone-induced cell death. Dendritic spines are known to change morphologically early in the process of neuronal death, which is known as dendritic beading [26,27]. This has been observed as a neuronal disorder in PD, and it has been reported that dendrites become shorter in the striatum of PD patients [28,29]. Furthermore, one sign of dendritic beading is shortened dendritic length. Thus, our finding that 1,5-AF treatment reversed the rotenone-induced dendritic shortening suggests that 1,5-AF has cytoprotective effects.

Furthermore, metformin is known to have mitochondrial protective activity [20]. In particular, metformin reportedly activates AMPK, which in turn activates mitochondrial biogenesis via PGC-1α. AMPK is a major activator of various molecules and is believed to be activated by phosphorylation [23]. Activated AMPK then activates PGC-1α via the activation of SIRT1 [30,31]. PGC-1α is activated by deacetylation [31,32], and activated PGC-1α causes mitochondrial biogenesis [33]. Mitochondrial biogenesis is the process by which cells increase the mass and copy number of individual mitochondria, thereby increasing ATP production. Metformin is known to activate AMPK in hepatocytes and muscle cells [34]. In human umbilical vein endothelial cells, metformin activates AMPK, increases expression of PGC-1α, and causes mitochondrial biogenesis [22]. Furthermore, metformin increases phosphorylated AMPK, increases expression levels of sirtuin 1 (SIRT-1) and PGC-1α, and causes mitochondrial biogenesis in C2C12 mouse skeletal muscle cells [23]. In the present study, treatment with metformin also led to increased dendritic length compared with rotenone-treated cells. However, the 1,5-AF metabolite 1,5-AG did not have this effect. Thus, although 1,5-AF is rapidly reduced to 1,5-AG in the kidney, the present study results suggest that this reduction does not occur rapidly in neuronal cell culture. In addition, a relatively high concentration of metformin had protective effects against rotenone-induced mitochondrial toxicity in PC12 cells in the present study. In contrast, a relatively low concentration of 1,5-AF had similar protective effects. These findings indicate that, compared with metformin, 1,5-AF may have greater therapeutic potential against PD. However, to our knowledge, there have been relatively few investigations regarding the metabolism and synthesis of 1,5-AF in the human body. In humans, potential clinical applications require in vivo analyses of 1,4-glucan lyase overexpression and the potential inhibition of 1,5-AF metabolism in the kidney.

In the current study, we also confirmed that 1,5-AF upregulated PGC-1α expression and enhanced its deacetylation, despite the presence of rotenone. Although rotenone downregulated the expression of PGC-1α, this effect was attenuated by co-treatment with 1,5-AF. Moreover, 1,5-AF treatment led to increased deacetylation of PGC-1α in vitro while inducing PGC-1α expression. 1,5-AF treatment also enhanced AMPK phosphorylation; therefore, 1,5-AF may activate PGC-1α in an AMPK-dependent manner leading to mitochondrial biogenic activity and cytoprotection. While these effects may be present in the absence of rotenone, they appear essential for protecting against rotenone-induced cytotoxicity. Additionally, 1,5-AF without rotenone treatment increased dendritic length and mitochondrial biogenesis, but it did not significantly increase PGC-1α protein levels. This may be related to the considerable variability in observed PGC-1α levels; nonetheless, these levels tended to increase without rotenone treatment.

The cytoprotective effects of 1,5-AF were inhibited when PGC-1α was silenced by *PPARGC1A* siRNA transfection. In addition, although 1,5-AF treatment led to increased MitoTracker intensity and activity of mitochondria, these effects were also inhibited when PGC-1α was silenced. Together, these findings suggest that PGC-1α is essential for both the cytoprotective and mitochondrial biogenic effects of 1,5-AF treatment. While we cannot rule out the possibility that 1,5-AF is involved in other unidentified pathways, the findings in *PPARGC1A* siRNA experiments suggest that the primary pathway is as follows: 1,5-AF activates AMPK leading to PGC-1α activation, which contributes to mitochondrial protection and proliferation. As mentioned above, these effects may be present in the absence of rotenone; they also appear to be essential for protection against rotenone-induced cytotoxicity. Future studies (based on our preliminary in vitro findings) should consider directly targeting PGC-1α levels and investigate the potential therapeutic effects of this protein in an in vivo model.

This study had some limitations. First, we could not confirm the in vitro effects in vivo because of regulations concerning rotenone handling in animal centers in Japan. To test the effects of 1,5-AF in vivo, we are currently developing a PD animal model using alternative methods. These methods include a Drosophila model, in which rotenone treatment can induce mitochondrial defects and dopaminergic neuronal loss [35]. Second, in the current study, rotenone and 1,5-AF were generally administered simultaneously. In PD in humans, however, cell loss begins long before therapeutics can be administered. Thus, future studies should explore the effects of 1,5-AF at a range of time points after rotenone exposure. Our preliminary analysis suggested a potential cytoprotective effect in PC12 cells exposed to rotenone for 1 h, followed by exposure to 1,5-AF in rotenone-free media for 24 h (*p* < 0.05, compared with rotenone alone; Supplementary Figure S5). These effects imply the potential for development and eventual application in the treatment of patients with current PD; to our knowledge, there is no oral medication that can fully reverse the effects of PD in clinical practice. Third, we could not test the effects of 1,5-AG and metformin in all of the presented experimental conditions. Concerning 1,5-AG, we were limited by financial constraints. At the same time, the effects of metformin in this study depended on the use of a high drug dose, which affected other experimental reagents and made measurements challenging. We suspect that these changes in experimental reagents may have been related to cellular acidosis, based on the side effect of lactic acidosis observed during the clinical application of metformin [36,37]. Notably, we excluded metformin from experiments other than those shown in Figure 4, Figure 5 and Figure 6 because those findings strongly implied that the effects of 1,5-AF were similar to those of metformin; this is consistent with a published approach [22]. In future studies, we will explore the cytoprotective effects of 1,5-AG in a more extensive range of experiments. Fourth, some assessments of cytoprotective effects (i.e., results in Figure 4) were performed using pooled data; the resulting variability among conditions may have influenced the strength of statistical comparisons. Fifth, mitochondrial membrane potential may be reduced upon rotenone administration; thus, the intracellular mitochondrial mass estimated by the MitoTracker assay may be lower under conditions of rotenone administration [38]. In future analyses of 1,5-AF, the mitochondrial mass should be investigated by using fluorescent probes such as nonyl acridine orange, for which mitochondrial uptake is unaffected by membrane potential [39].

In conclusion, 1,5-AF inhibited rotenone-induced cytotoxicity in a range of cell lines in the present study. These cytoprotective effects of 1,5-AF likely involved mitochondrial biogenesis and were dependent on the transcription coactivator PGC-1α. Together, these findings suggest that 1,5-AF has therapeutic potential as a treatment for PD and should be further investigated.

## 4. Materials and Methods

### 4.1. Materials

Unless otherwise specified, all reagents used in this study were purchased from Sigma-Aldrich (St. Louis, MO, USA). The pure 1,5-AF was produced by SUNUS Corporation (Kagoshima, Japan) from sweet potatoes using an enzyme, α-1,4-glucan lyase (EC 4.2 2.13), as previously reported [40]. The 1,5-AG was obtained from FUJIFILM Wako Pure Chemical Corporation (Osaka, Japan). Poly-D-lysine four-well culture slides were obtained from Biocoat, Inc. (Horsham, PA, USA). MTT assay kits were purchased from Dojindo Laboratories (Kumamoto, Japan). Mitochondrial ToxGlo™ Assay kits to measure intracellular ATP were purchased from Promega Corporation (Madison, WI, USA). The antibodies against β-actin (cat. # A1978) and MAP2 (cat. M4403, Sigma-Aldrich (St. Louis, MO, USA)) were obtained from Sigma-Aldrich; corresponding secondary antibodies were obtained from Santa Cruz Biotechnology, Inc. (Santa Cruz, CA, USA) and Biotium (Hayward, CA, USA), respectively. The antibody against PGC-1α was obtained from Novus Biologicals (cat. # NBP1-04676; Littleton, CO, USA). Antibodies against ACC-Ly (cat. # 9441), AMPK (cat. # 2532), and phospho-AMPK (cat. # 2535) were purchased from Cell Signaling Technology (Danvers, MA, USA). Calcein AM double staining kits and Hoechst 33342 were purchased from Dojindo Laboratories (Kumamoto, Japan).

PC12 cells, which are derived from rat pheochromocytoma, were used for the majority of the cell culture experiments because they have been used previously as an in vitro neuronal cell model [41]. These cells have also been used to explore the mechanisms of dopaminergic cell degeneration in PD [42].

Rat PC12 cells [43] were obtained from the American Type Cell Culture Collection (Manassas, VA, USA). The cell lines were maintained in RPMI-1640 with 10% fetal bovine serum, 100 U/mL penicillin, and 100 mg/mL streptomycin (Invitrogen, Grand Island, NY, USA) on poly-L-lysine-coated dishes at 37 °C in a humidified 5% CO_2_ atmosphere.

Mouse primary cortical and hippocampal neurons, derived from the cerebral cortex, were obtained from Dr. Yuki Kambe, Department of Pharmacology, Kagoshima University Graduate School of Medical and Dental Sciences. We used C57BL6/J mice, which were purchased from Japan SLC Inc. (Shizuoka, Japan). Mouse primary neurons were cultured as previously described [44]. Briefly, fetal cerebral cortices were removed on day 14 of gestation and treated with trypsin. The dispersed cells were stored in liquid nitrogen. Next, the cells were seeded at a density of 30,000 cells/cm^2^ on poly-L-lysine-coated culture plates. The cells were cultured in Neurobasal/B27 medium supplemented with 500 µM glutamine and 25 µM glutamate at 37 °C in a humidified 5% CO_2_ atmosphere. On day 2, the cells were treated with 1 µM cytosine arabinoside (Ara-C). On day 4, cells were washed twice in phosphate-buffered saline (PBS); the medium was then replaced with Neurobasal/B27 medium supplemented with glutamine only (not glutamate). On days 14 to 21, the cell stimulation experiments were performed.

All confocal images were taken using the Keyence BZ-X700 All-In-One Fluorescence Microscope (Keyence Co., Osaka, Japan) and analyzed using BZ-X Analyzer version 1.3.1.1 (Keyence Co.) or ImageJ version 1.51k software (National Institutes of Health, Bethesda, MD, USA).

All experiments were performed in triplicate except for those shown in Figure 4 and Figure 5 (quadruplicate) and in Figure 7c and Figure 8a–d (duplicate).

### 4.2. Cell Stimulation

PC12 and mouse primary neuronal cells were used in this study. To investigate the toxicity of rotenone to PC12 cells, we dissolved rotenone in DMSO at final concentrations ranging from 0 to 100 μM, then exposed the cells to this dissolved rotenone for 24 h, based on previous reports [45,46]. The concentration of rotenone at which 50% of PC12 cells survived was 1 μM (Supplementary Figure S1). In experiments with primary mouse neuronal cells, rotenone was dissolved in DMSO at a concentration of 50 nM, based on previous reports [45,46]. Monolayer cells were then treated with or without rotenone and with or without either 1,5-AF (5–100 μg/mL), 1,5-AG (5–100 μg/mL), or metformin (1.25 mM) for 24 h.

### 4.3. MTT Assay to Analyze Cell Viability

PC12 or mouse primary neuronal cells were seeded in 96-well plates (2 × 10^4^ cells per well) and incubated with DMSO or rotenone (1 µM) for 24 h. The numbers of live cells were then evaluated using the MTT assay, which was performed in accordance with the manufacturer’s instructions. Cell numbers were assessed using the Keyence BZ-X700 All-In-One Fluorescence Microscope and BZ-X Analyzer software.

### 4.4. Anti-MAP2 Immunofluorescence Staining

The protective activity of 1,5-AF against the cytotoxic effects of rotenone was evaluated on the basis of dendritic morphology and length. Dendrites were visualized by staining the cells with an anti-MAP2 antibody. Briefly, primary neuronal cells were plated in poly-L-lysine-coated 48-well dishes. Cells were fixed for 20 min with 4% paraformaldehyde in PBS, washed with PBS, and incubated in blocking solution for 20 min. Cells were then incubated with antibodies specific for MAP2, followed by incubation with secondary antibodies coupled to CF488. Next, cells were washed with PBS, then stained with 0.25 µg/mL Hoechst 33342 in PBS. Finally, the cells were analyzed using ImageJ.

### 4.5. Immunoblotting

Cell monolayers were stimulated by 1 µM rotenone for 24 h with or without 1,5-AF (50 µg/mL) or metformin (1.25 mM). The medium was then washed out, and the monolayers were rinsed using saline. For the detection of PGC-1α and other proteins, immunoblotting was performed. Briefly, cell lysates were diluted 1:1 in 2× sample buffer (100 mM Tris-HCl pH 6.8, 4% sodium dodecyl sulfate, 10% 2-mercaptoethanol, 20% glycerol, and 0.01% bromophenol blue) and boiled for 5 min. Next, 5 µg of protein from each lysate was separated on a polyacrylamide gel, blotted on a polyvinylidene fluoride membrane, and probed with antibodies against PGC-1α and the other proteins. Note that, to detect the percentage of phospho-AMPK and AMPK, the proteins were simultaneously run on separate gels and normalized using antibodies against β-actin [21]. Horseradish peroxidase-conjugated anti-rabbit or anti-mouse IgG polyclonal antibodies were used as secondary antibodies. Bands were visualized using an enhanced chemiluminescence detection system (Millipore, Burlington, MA, USA) and measured using ImageJ software.

### 4.6. PGC-1α Acetylation Assays

Evaluation of acetylation of PGC-1α was detected by immunoprecipitation [22], based on a report that an increased level of ACC-Ly implies a decreased level of deacetylated PGC-1α [47]. In accordance with a published protocol [22], PGC-1α protein was immunoprecipitated with anti-PGC-1α antibody (Novus Biologicals) and agarose beads (Santa Cruz); PGC-1α levels and acetylation were then detected using specific antibodies for PGC-1α (Novus Biologicals) and ACC-Ly (Cell Signaling), respectively.

### 4.7. Silencing of PGC-1α Using PPARGC1A siRNA for the MTT Assay

PC12 cells (1 × 10^5^/mL) were cultured for 24 h in four-well chamber dishes (Cosmo Bio, Tokyo, Japan) before the following experiments were conducted. The cells were transfected with 1 pmol/well of control or *PPARGC1A* siRNA (Thermo Fisher Scientific, Waltham, MA, USA) using Lipofectamine RNAiMAX (Thermo Fisher Scientific), then cultured in medium containing D-(+)-galactose instead of D-glucose for the appropriate period, based on a published protocol [48].

For the MTT assay, cells (1 × 10^5^ per well) were incubated with RPMI-1640 medium containing 10% fetal bovine serum for 48 h. Next, 1/16 diluted Lipofectamine RNAiMAX Reagent (Thermo Fisher Scientific) in Opti-MEM Medium (Thermo Fisher Scientific), and 1/50 diluted siRNA in Opti-MEM Medium were prepared. The diluted siRNA was added to the diluted Lipofectamine RNAiMAX Regent (1:1 ratio). Next, the medium in the dishes was replaced with RPMI-1640 and 2% fetal bovine serum, and the siRNA–lipid complex was added to cells (100 mL/well) for 5 min at room temperature.

### 4.8. Analysis of Mitochondrial Mass by MitoTracker

Rat PC12 cells were cultured on four-well chamber dishes (Cosmo Bio). The medium was changed to RPMI-1640 with 2% fetal bovine serum. Subsequently, DMSO alone or rotenone (final concentration, 1 µM) dissolved in DMSO was added to each well; cells were then treated with PBS alone or 1,5-AF (final concentration 50 µg/mL) dissolved in PBS for 24 h. Next, the medium was changed to phenol red-free RPMI-1640 with 2% fetal bovine serum; cells were incubated with MitoTracker red (CMH2XROS, Thermo Fisher Scientific; final concentration, 1 µM) and Hoechst 33342 for 1 h. Cells were washed with warmed PBS and fixed in 4% formaldehyde. The fixed cells were examined under a fluorescence microscope (BZ-X700, Keyence Co.). Images were acquired using the same exposure time in each well. The experiment was performed in triplicate. Four images were extracted from the same well. In addition, five cells were selected from each image, the area and intensity were measured by ImageJ, and the mean values for each image were calculated. To normalize the MitoTracker staining results, the area density values of the fluorescent images in each group were divided by the area density values of the fluorescent images in the DMSO + PBS group (i.e., the normal control group).

### 4.9. Intracellular ATP Assay

ATP produced in the wells was measured using the Mitochondrial ToxGlo Assay kit (Promega Corporation). Cell medium was replaced with RPMI-1640 with D-(+)-galactose instead of glucose. The assay was then performed in accordance with the manufacturer’s protocols. For normalization of ATP assay results, the background absorbance value was subtracted from each recorded absorbance value; the resulting value was then divided by the absorbance value of the DMSO + PBS group (i.e., the normal control group).

### 4.10. Statistical Analysis

All data are expressed as the mean ± standard error of the mean. Unless otherwise specified, the results were analyzed using the unpaired *t*-test to determine the statistical significance of the treatment sets. Differences were considered statistically significant when *p* < 0.05.

## Data Availability

The data that support the findings of this study are available from the corresponding author, Ikuro Maruyama, upon reasonable request.

## Supplementary Materials

Supplementary materials are available online at https://www.mdpi.com/article/10.3390/ijms22189941/s1.
