# Supplementary material for: 1,5-Anhydro-D-fructose Protects against Rotenone-Induced Neuronal Damage In Vitro through Mitochondrial Biogenesis
Source: Int J Mol Sci. 2021 Sep 14;22(18):9941. doi: 10.3390/ijms22189941 (PMC8466044; doi:10.3390/ijms22189941)
Supplement: Supplementary file 1 [file ijms-22-09941-s001.zip › ijms-1374549-SI.pdf]

Supplementary Materials

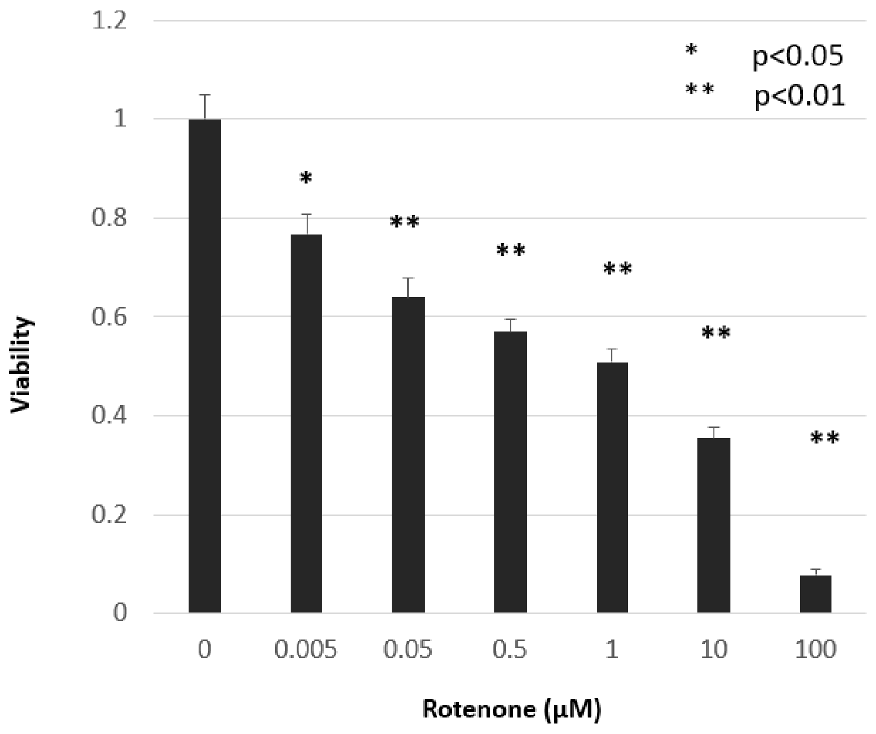

Supplementary Figure S1. Rotenone-induced cytotoxicity in cultured PC12 cells. Cells were incubated with control solvent (dimethyl sulfoxide) or rotenone for 24 hours; viable cells were then counted to evaluate the cytotoxic effects of rotenone in this cell line (one-way analysis of variance). Rotenone was cytotoxic in a dose-dependent manner. All data are expressed as the mean ± standard error of the mean. *$p < 0.05$, **$p < 0.01$.

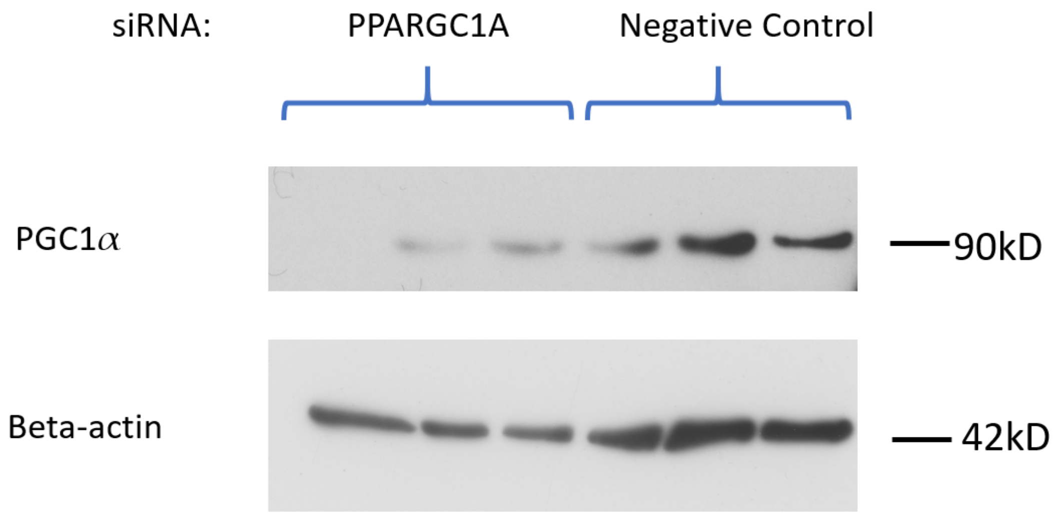

Supplementary Figure S2. PGC-1α protein levels were assessed by immunoblotting. PGC-1α protein was reduced by transfection with *PPARGC1A* siRNA, which silences PGC-1α expression. PGC-1α, peroxisome proliferator-activated receptor-γ coactivator 1α; siRNA, small interfering RNA.

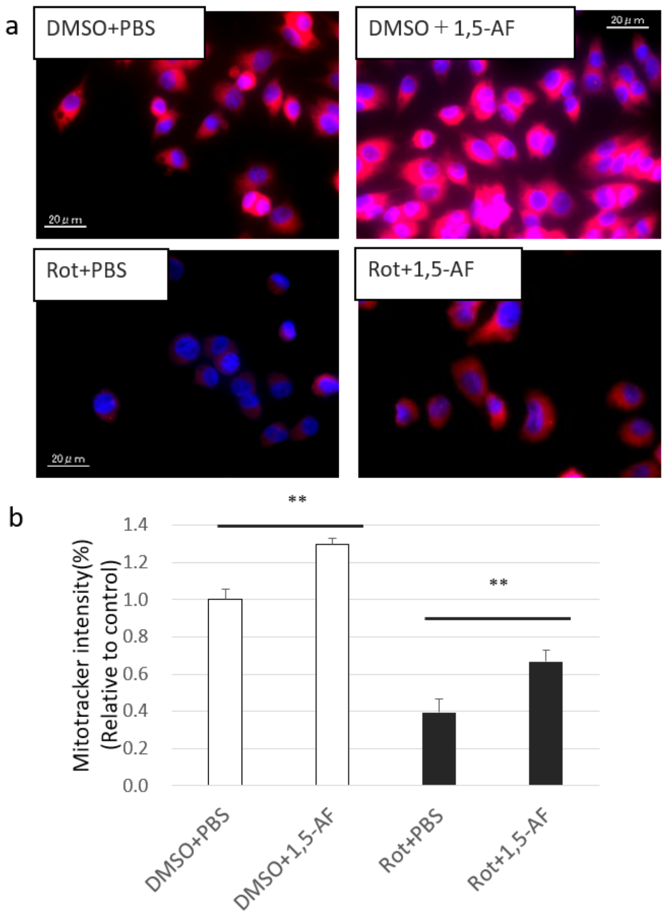

Supplementary Figure S3. Effects of 1,5-AF treatment on mitochondrial quantity and quality in cultured PC12 cells. (a) Representative confocal images of MitoTracker staining (magnification: 100×; scale bar: 20 μm). (b) Quantification of the effects of 1,5-AF treatment on MitoTracker intensity, from confocal images of MitoTracker-stained cells. All data are expressed as the mean ± standard error of the mean. *$p < 0.05$, **$p < 0.01$. 1,5-AF, 1,5-anhydro-D-fructose; DMSO, dimethyl sulfoxide; PBS, phosphate-buffered saline; Rot, rotenone.

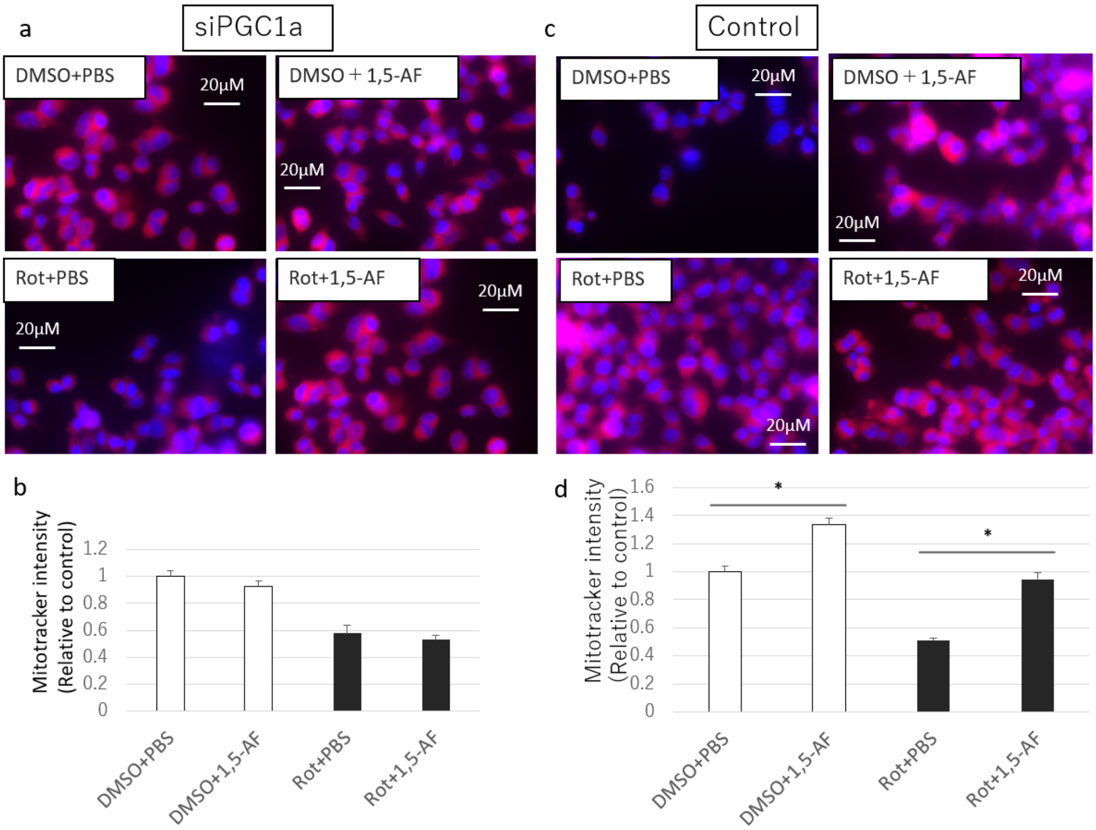

Supplementary Figure S4. Effects of PGC-1α silencing on the mitochondrial protective activity of 1,5-AF against rotenone treatment in cultured PC12 cells. (**a**) Representative confocal images of MitoTracker staining in cells transfected with *PPARGC1A* small interfering RNA (siRNA; magnification: 100×, scale bar: 20 μm). (**b**) Transfection with *PPARGC1A* siRNA inhibited the increase in MitoTracker intensity of 1,5-AF treatment in rotenone-treated cells. (**c**) Representative confocal images of MitoTracker staining in cells transfected with control siRNA (magnification: 100×; scale bar: 20 μm). (**d**) In cells transfected with control siRNA, treatment with 1,5-AF increased the MitoTracker intensity in both DMSO- and rotenone-treated cells. All data are expressed as the mean ± standard error of the mean. *$p < 0.05$. 1,5-AF, 1,5-anhydro-D-fructose; DMSO, dimethyl sulfoxide; PBS, phosphate-buffered saline; Rot, rotenone; siPGC1a, cells transfected with *PPARGC1A* siRNA.

R10uM 1h + stim 24h

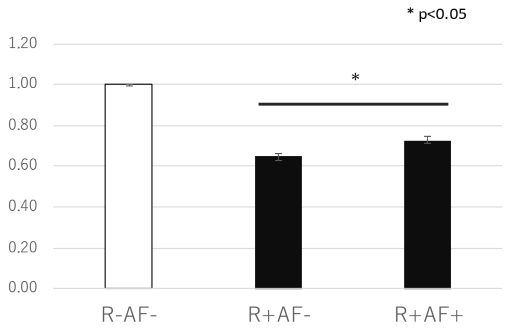

Supplementary Figure S5. Protective effects of 1,5-AF in PC12 cells exposed to rotenone prior to 1,5-AF treatment, evaluated using the MTT assay. Cells were cultured without rotenone (10 μM) or 1,5-AF for 25 hours (R-AF-), with rotenone (10 μM) for 1 hour and with rotenone-free media for 24 hours (R+AF-), or with rotenone (10 μM) for 1 hour and with 1,5-AF (50 μg/mL) in rotenone-free media for 24 hours. All data are expressed as the mean ± standard error of the mean of triplicate experiments. *$p < 0.05$, **$p < 0.01$. 1,5-AF, 1,5-anhydro-D-fructose; R, rotenone; stim, stimulation.

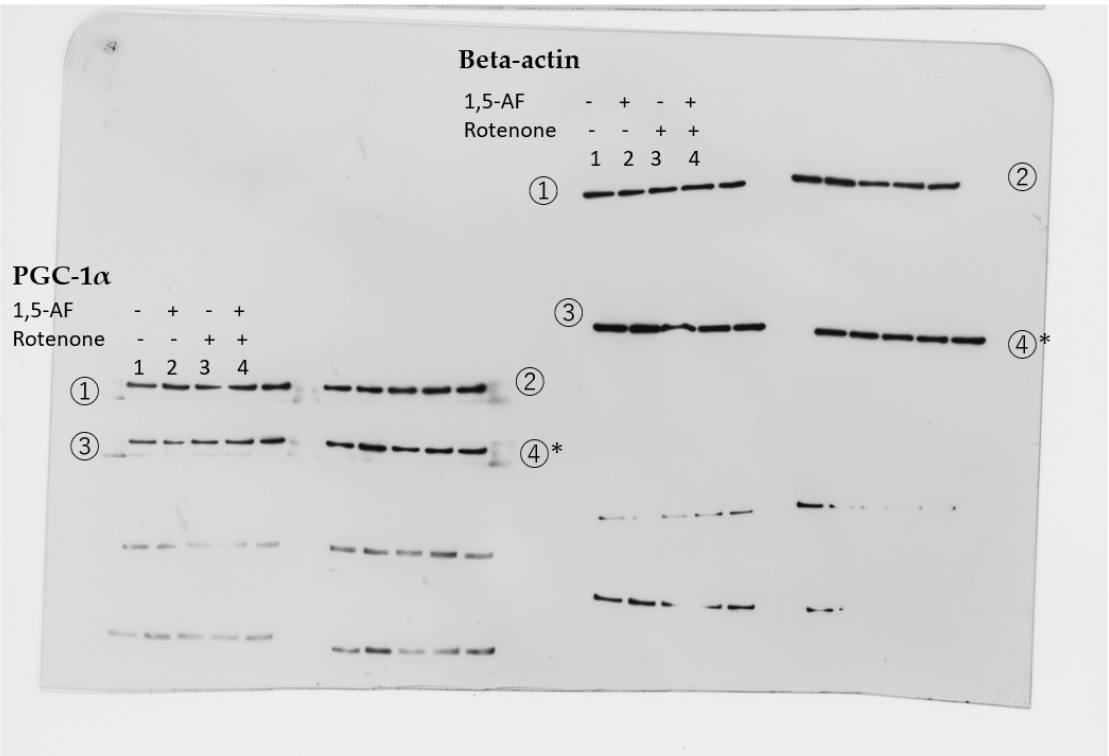

Supplementary Figure S6. Original scan data from Figure 5a. *Denotes the blot used in the figure. PGC-1α, peroxisome proliferator-activated receptor-γ coactivator 1α; 1,5-AF, 1,5-anhydro-D-fructose.

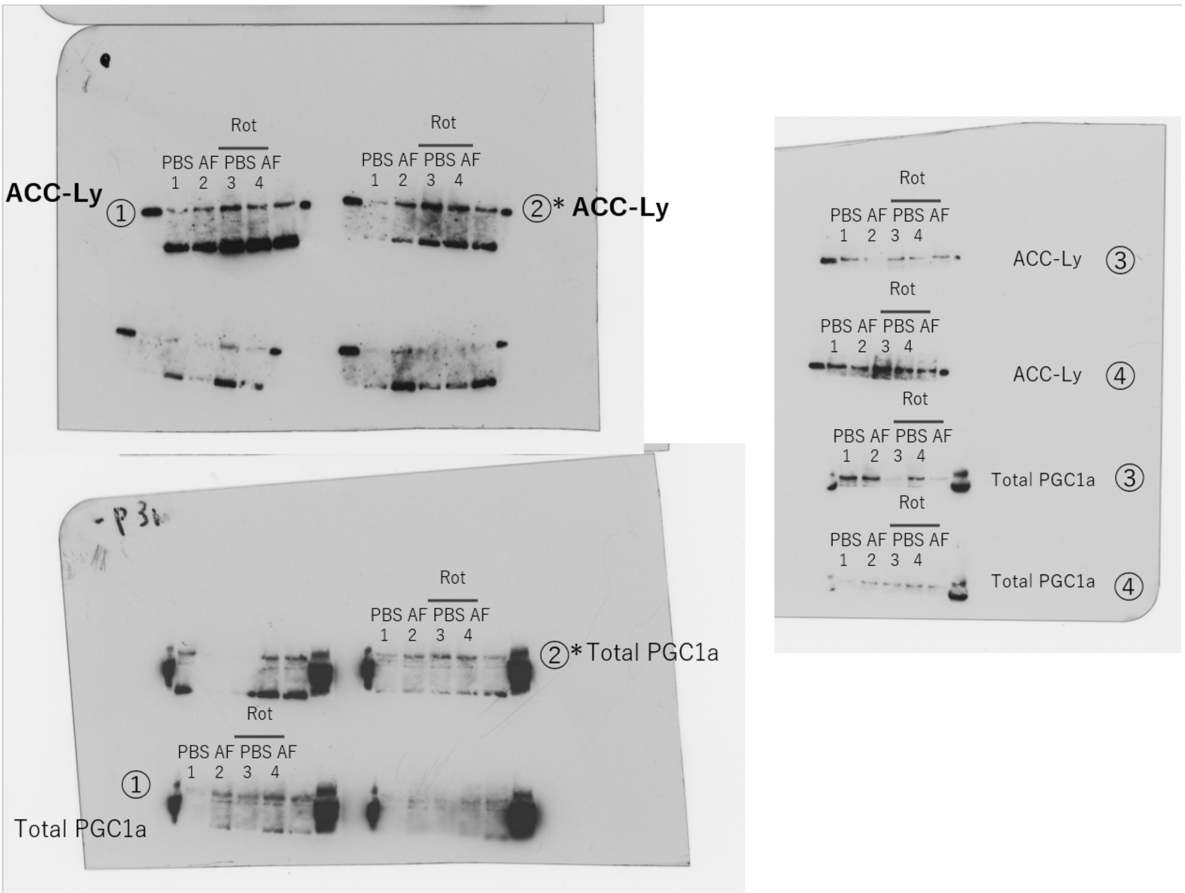

Supplementary Figure S7. Original scan data from Figure 5b. *Denotes the blot used in the figure. AF, 1,5-anhydro-D-fructose; Rot, rotenone; PBS, phosphate-buffered saline; ACC-Ly, acetylated lysine; PGC-1a, peroxisome proliferator-activated receptor-γ coactivator 1α.

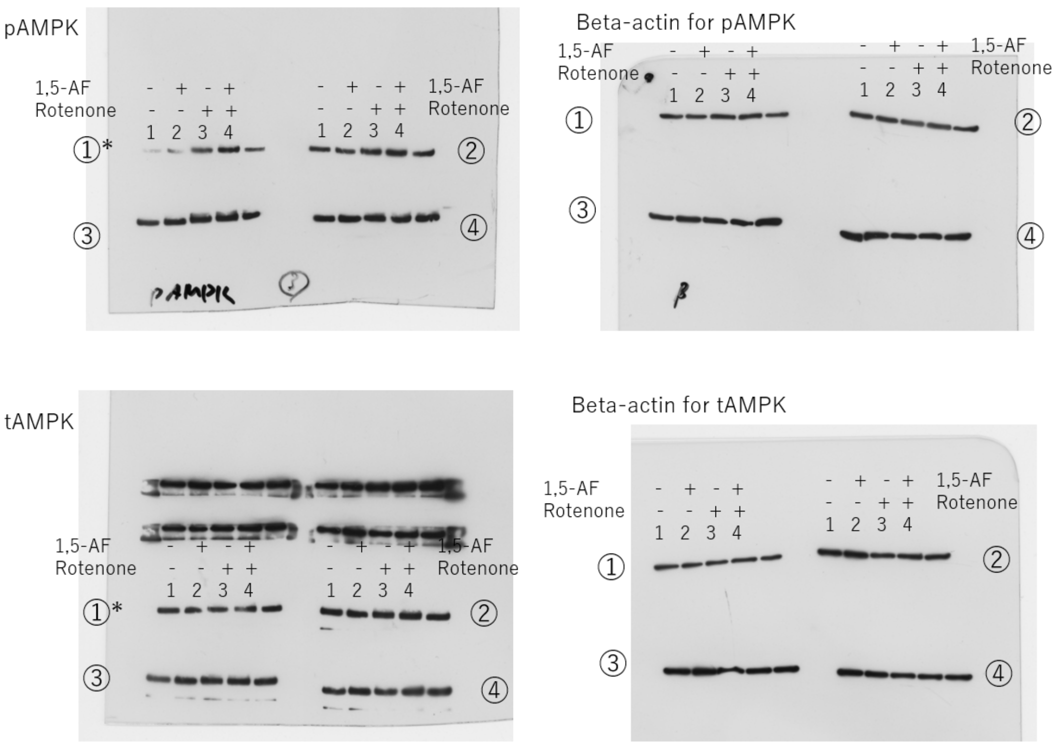

Supplementary Figure S8. Original scan data from Figure 5c. *Denotes the blot used in the figure. 1,5-AF, 1,5-anhydro-D-fructose; pAMPK, phosphorylated AMP-activated protein kinase; tAMPK, total AMP-activated protein kinase.
